# Toward an autism-friendly environment based on mobile apps user feedback analysis using deep learning and machine learning models

**DOI:** 10.7717/peerj-cs.1442

**Published:** 2023-08-09

**Authors:** Mariem Haoues, Raouia Mokni

**Affiliations:** 1 Department of Software Engineering, College of Computer Engineering and Sciences, Prince Sattam bin Abdulaziz University, Al-Kharj, Saudi Arabia; 2 Université de Carthage, Faculté des Sciences de Bizerte, Bizerte, Zarzouna, Tunisia; 3 Department of Information System, College of Computer Engineering and Sciences, Prince Sattam bin Abdulaziz University, Al-Kharj, Saudi Arabia; 4 University of Gabès, Higher Institute of Management of Gabès, Gabès, Tunisia

**Keywords:** Autism spectrum disorder, Autism-friendly environment, User feedback, Mobile apps, Deep learning models, RNN, LSTM, Sentiment analysis

## Abstract

Autistic people are often disadvantaged in employment, education, *etc*. In fact, autistic students/employees face several challenges navigating and communicating with their superiors and colleagues. Mobile applications for people with Autism Spectrum Disorder (ASD apps for short) have been increasingly being adapted to help autistic people manage their conditions and daily activities. User feedback analysis is an effective method that can be used to improve ASD apps’ services. In this article, we investigate the usage of ASD apps to improve the quality of life for autistic students/employees based on user feedback analysis. For this purpose, we analyze user reviews suggested on highly ranked ASD apps for college students, and workers. A total of 97,051 reviews have been collected from 13 ASD apps available on Google Play and Apple App stores. The collected reviews have been classified into negative, positive, and neutral opinions. This analysis has been performed using machine learning and deep learning models. The best performances were provided by combining RNN and LSTM models with an accuracy of 96.58% and an AUC of 99.41%. Finally, we provide some recommendations to improve ASD apps to assist developers in upgrading the main services provided by their apps.

## Introduction

### Mobile apps for autistic

Autism Spectrum Disorder (ASD) is a “neurological and developmental disorder characterized by severe and sustained impairment in social interaction, deviance in communication, and patterns of behavior and interest that are restricted, stereotyped, or both” (Kanner, 1971). According to the 1st edition of the Diagnosing the Diagnostic and Statistical Manual of Mental Disorders (Cooper, 2018), ASD is defined as a status described by deficits in two main fields: (1) social communication and social interaction, and (2) restricted repetitive patterns of behavior, interests, and activities. Recent studies proved that ASD could be reliably diagnosed when the child is about 14 to 18 months old (*cf.*, Hogan et al., 2020; Mokni & Haoues, 2020). In the last few years, the number of people with autism has rapidly increased, where, about 1 in 36 children being Autistic according to estimates from Centers for Disease Control’s Autism and Developmental Disabilities Monitoring (ADDM) Network (Maenner et al., 2023). In 2017 and specially in Saudi Arabia, the number of individuals diagnosed with autism was 42,500, and many remained undiagnosed (Khan et al., 2020).

In third-world countries, providing special education for autistic has been slow. This is affected by the individual’s culture and attitude (Sulaimani & Gut, 2019). People with autism need special care and services, however, the increasing number of individuals being diagnosed with autism made things more challenging. In fact, providing services and treatments became demanding and stressful for family members and service providers, including special education professionals. In particular, college students and workers with ASD might experience several communication issues, which make their daily activities challenging. Moreover, the environment influences people with ASD behavior such as loud noises, strong smells, *etc*.

Recent research studies proved that there is an important gap between the number of autistic students and non-autistic students, this gap is also represented in employment. For instance, in the United Kingdom, 30% of graduates with autism are in full-time jobs compared to 70% of graduates without disabilities (Pesonen et al., 2021). In fact, autistic people have a lower functional status in performing daily tasks, while the majority need assistive technologies. In addition, people with ASD struggle in communicating with others, sensory sensitivities, understanding social interaction, and even developing language. These difficulties can make universities/companies’ access and interactions challenging. For these reasons, students and employees with autism need extra care to succeed in their studies and work. Nonetheless, with the common communication and technology evolution growth, new technologies such as mobile apps provide assistance for people with ASD, their families, their co-workers, *etc*.

Over the last years, Information and Communication Technology is increasingly being integrated into the healthcare industry. Recently, various research and development projects involved the use of new technologies (*e.g*., smartphones, wearables, *etc*.) to support the different stakeholder’s needs (*e.g*., patients, healthcare professionals, *etc*.). Several mobile apps are provided to help people with autism and their families survive daily challenges, enhance learning, and educate themselves on autism. These apps are used for education, communication, behavior, and sensory issues. Based on our research conducted in August 2022, the total number of ASD apps available on the Google Play and Apple App stores are respectively 248 and 128 apps. They are used by individuals with autism aged from 2 years old to adults, their parents, and healthcare professionals, however, the majority of these apps are developed for children, usually, they are formatted to be played as games.

### User feedback analysis

User feedback provides information delivered by users to express their opinions towards a specific product/service if they are satisfied or dissatisfied with it. User feedback is valuable for two main reasons: firstly, user reviews help in determining product issues and enhancing product quality (de Lima, de Araújo & Marcacini, 2022). Secondly, other users consider reviews a reliable source of information, so reading negative feedback could alienate potential users from trying the product/service. The purpose of any software organization is to design and deliver platforms that fulfill and satisfy user needs to attract more users. The continuous update is a factor in the platform’s success. Besides, in every update, it is compulsory for software developers to investigate the previous version issues, and the common traditional way is software testing, which tends to be cumbersome, labor-intensive, and time-consuming in practice (Gao et al., 2019).

Google Play and Apple App stores allow users to rate and submit their experience of any platform they used publicly. Software developers have access to thousands of user reviews that could help them identify apps’ issues. Thus, user reviews could help identify the possible improvement paths of mobile apps and determine the shortfalls of the current version. For this reason, the user feedback analysis method gains success lately in the software maintenance practice.

### Objectives and article organization

Recent research studies investigated the use of telehealth services to improve the quality of life for people with autism. The main objective of this article is to provide key insights to improve mobile apps for people with ASD based on user feedback analysis using machine learning and deep learning models. The selected apps in this article will focus on college students and workers as primary users. In fact, throughout our conducted research, we noticed that the number of mobile apps destined for this category is very limited in comparison to the number of mobile apps developed to be used by autistic children. Hence, the main contributions of this research work are listed as follows:

- Firstly, we analyze the selected ASD apps to identify the main features provided for college students and workers with ASD.
- Second, we propose to use machine learning and deep learning techniques for user feedback analysis on the selected ASD apps. For this purpose, we classify user reviews into positive, negative, and neutral opinions.
- Third, we identify the main challenges of ASD apps and suggest some improvements to assist developers in improving the features provided by their apps.

The work presented in this research article is new compared to the literature review. In fact, to the best of our knowledge, there is no similar work that addressed the analysis of user feedback to evaluate the features provided by ASD apps. In addition, this article focuses on college students, and workers with ASD as primary users of the selected apps. The rest of this work is presented as follows: the “Related work” section reviews some previous work that used machine learning and deep learning techniques to improve mobile apps’ features. In the section “Research Work Process”, we present our adapted research work process. Section “Mobile apps for ASD” applies the Systematic Literature Review (SLR) process to evaluate mobile apps for ASD. In the section “Machine Learning and Deep Learning for user feedback analysis”, we use machine learning and deep learning to analyze user feedback on ASD apps. Section “Experimental Results” presents the obtained results. In section “Challenges and recommendations for ASD apps”, we suggest possible paths for the improvement of ASD apps and discuss some limitations of this study. Finally, the “Conclusions” section summarizes the presented work and outlines some of its possible extensions.

## Related work

Autism Spectrum Disorder (ASD) is a biological-based neurodevelopmental disorder that is recognized in childhood. This disorder has several symptoms, some of which are more severe than others. In this section, we initially review previous studies that investigated the importance of technologies in the autistic lifestyle. Then, we present some research work that used machine learning or deep learning models to improve mobile apps.

### Technologies and autistic special needs

Assistive technologies help autistic people increase their independence with daily living and mobility, improve their communication skills, *etc*. For instance, O’Brien et al. (2020) developed several techniques to help students with ASD join their classmates to learn without any difficulties using a smartwatch. The main purpose of these techniques is to allow students with ASD to use the same technologies as their colleagues. Smartwatches are used to provide directions for students with ASD, remind them to do homework, facilitate interactions with their classmates, *etc*. This will guarantee the student’s independence. On the other hand, Gurbuz, Hanley & Riby (2019) used an online questionnaire to investigate the social and academic experiences of autistic university students in the United Kingdom. The results reported in this research study highlighted the several challenges and difficulties that are faced by an autistic student, such as mental health issues, social skills, autism awareness from others, *etc*. In contrast, several strengths regarding the academic skills of autistic students have been noticed as well. Finally, Pesonen et al. (2021) conducted interviews with 30 autistic university students from Finland, France, the Netherlands, and the United Kingdom to evaluate the support offered by universities for autistic students to help them in their transition to the workspace. The results of this study reported that the support offered by these universities does not satisfy the students’ needs.

### Improving mobile apps using machine learning and deep learning models

User feedback review is considered a beneficial solution to improve mobile apps with respect to users’ needs. For this purpose, researchers applied several techniques to automatically analyze and evaluate user reviews in order to detect which feature needs to be improved in the next update of their apps. Some researchers used machine learning models to review user feedback. For instance, Oyebode, Alqahtani & Orji (2020) evaluated the mental health apps by performing a sentiment analysis on user reviews using five machine learning classifiers: support vector machine (SVM), multinomial naïve bayes (MNB), stochastic gradient descent (SGD), logistic regression (LR), and random forest (RF). SGD was the most performing with an 89.43% accuracy score. They also conducted a thematic analysis of positive and negative reviews to identify different themes and factors that affect the apps’ performances. This study identified 21 negative factors (*e.g*., usability issues, content issues, ethical issues, *etc*.) and 29 positive factors (*e.g*., aesthetically pleasing interface, app stability, personalized content, *etc*.). In addition, Camacho-Rivera et al. (2020) proposed to evaluate the asthma mobile apps based on their provided features using the sentiment analysis of user reviews. For this purpose, the authors proposed to collect 373 user reviews from 10 apps for asthma, then they classified them into: high average rating and low average rating. Based on the analysis of user reviews, the authors revealed the important features provided by the highly ranked asthma apps such as tracking of peak flow readings and mentioned that usually the users complaint about the loss of data, and apps crashing. Moreover, Ossai & Wickramasinghe (2023) proposed to predict the users’ sentiments on diabetes mobile apps using embedded deep neural networks, K means, and Latent Dirichlet Allocation (LDA) techniques. The authors collected 38,640 comments from 39 diabetes mobile apps. After analyzing the user feedback the authors revealed that the main issues with diabetes mobile apps are their security, complexity in terms of interface, and their outdated information about this disease. In addition, the authors mentioned that the users are satisfied with some features provided by diabetes mobile apps such as the management of lifestyle, communication effectiveness, *etc*. On the other hand, Aslam et al. (2020) proposed an approach for the classification of app reviews based on the deep learning model into four categories: bug reports, enhancement reports, user experiences, and ratings. The proposed approach is evaluated using machine learning classifiers such as naïve Bayes (NB), MNB, decision tree (DT), SVM, convolutional neural network (CNN), and long short-term memory models. The obtained results showed that the best performances are provided by the CNN model with respectively 95.49%, 93.94%, and 94.71% for the averages of precision, recall, and F1-score measures. Finally, the authors suggested recommendations to improve the identified negative factors to better satisfy the user needs.

In summary, our literature review proved that several research studies (*cf.*, O’Brien et al., 2020; Gurbuz, Hanley & Riby, 2019; Pesonen et al., 2021, *etc*.) investigated the challenges that have been faced by autistic students and their educators. These studies revealed that some issues could be due to communication problems between students, educators, and university staff. On the other hand, autistic workers face several workplace challenges such as time management and organizational skills. The research study conducted by Bury et al. (2021) proved that adults with autism are significantly underrepresented in the workforce. The main workplace challenges faced by autistic workers revealed by Bury et al. (2021) could be characterized as (i) issues for autistic workers, and (ii) issues for their superiors. The discrimination in workplaces is due mainly to social differences, which leads to varying difficulties in understanding autistic and predicting their reactions. Mobile apps; such as the “Work Autonomy” app; are used to facilitate communication between autistic workers, their superiors, and their colleagues. Besides the social differences, autistic workers also have time-management problems. On the other hand, previous research studies proved that machine learning and deep learning models have been successfully used to improve mobile app features in order to better satisfy user needs.

On the other hand, researchers investigated the main features provided by ASD apps. For instance, Rehman et al. (2021) followed the PRISMA methodology to review the main features provided by 25 ASD apps such eye tracking, text-to-speech, *etc*. Based on the analysis of these features, the authors suggested some recommendations by adding Artificial Intelligence (AI) techniques for image recognition, progress tracking, *etc*. The successful application of AI-based technologies such as machine learning in diseases diagnosis encouraged the authors to propose their integration in ASD apps, however, the problem mentioned by the authors is the complexity and amount of data for people with autism, which is rarely recorded. While, for an accurate application of machine learning or deep learning models, an important amount of data is required. In addition, several families and professionals are increasingly using ASD apps for education. The study conducted by Gallardo-Montes, Caurcel Cara & Rodríguez Fuentes (2022) evaluated 155 apps selected from Google Play store to identify the main areas of ASD apps. This study identified that the majority of the selected apps focused on communication, emotions, time management and entertainment, but an improvement of their provided functionality is required.

## Research work process

**Figure 1:**
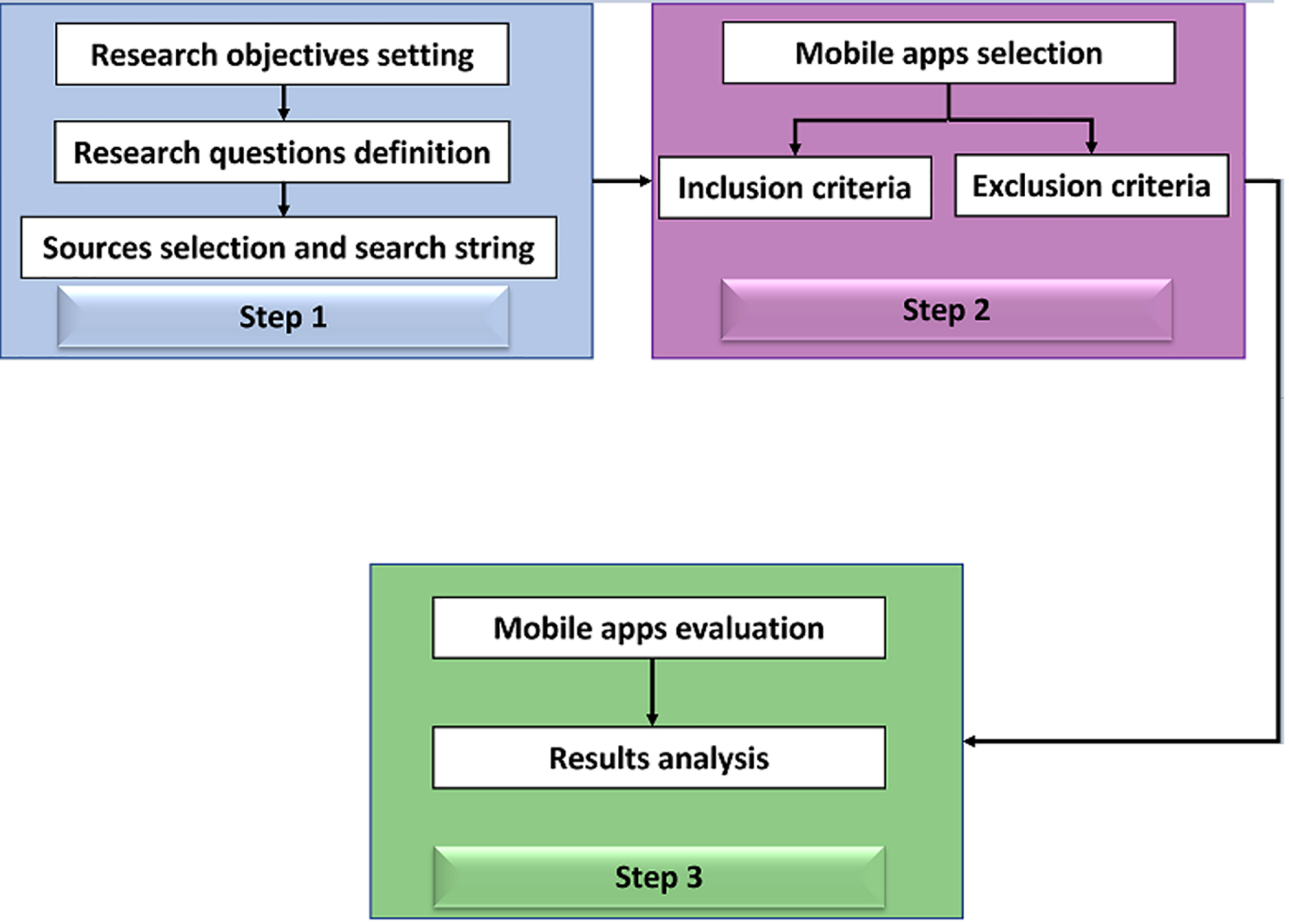
Systematic literature review process.

The systematic literature review (SLR) process, proposed by Kitchenham (2004), will be used to evaluate ASD apps (see Fig. 1). SLR has been successfully used previously in many research studies for ADS, such as evaluating the impact of technologies on autistic (Valencia et al., 2019), difficulties of transition to higher education for autistic students (Nuske et al., 2019), *etc*. Kitchenham (2004) summarized three essential stages to proceed with a literature review as follows:

- **Step 1:** It involves the setting of the research objectives and the definition of the research questions. Based mainly on these research questions, we searched for the mobile apps to be used in this study on Apple App store and Google Play store to collect the different ASD apps available on the market. Our search focused on ASD apps using search terms such as “autism”, and “autism spectrum disorder;”
- **Step 2:** In this step, we will use a set of inclusion and exclusion criteria to select the mobile apps to be used in our study from all the collected apps in Step 1;
- **Step 3:** This step involves the selected ASD apps evaluation to answer the research questions.

In this article, two research questions have been established to respond to the main objective of this study.

- **RQ1:** Does ASD apps assist adults with ASD to improve the quality of their lives?
- **RQ2:** Based on the user feedback, is there any service that needs to be improved?

## Mobile apps for asd

### Application sources and search terms

To proceed in this SLR, we searched for the most relevant ASD apps in the market based on two major sources: the Apple App store and the Google Play store. In fact, mobile apps in these two stores are classified into categories (*e.g*., Education, Health & Fitness, Medical, *etc*.). ASD apps have been compiled to help people living with autism and their families navigate daily challenges, enhance learning and educate themselves on autism.

To search for the ASD apps that will be used in this article, we used two keywords: “autism spectrum disorder” and “autism”. The selected terms were applied to the title and the description of the app. This research took place in August 2022. The total number of ASD apps available on the Google Play store is 248, whereas, only 128 ASD apps are available in the Apple App store. A total of 26 ASD apps are available in both stores. Hence, the total number of ASD apps initially collected is 350.

### Eligibility criteria and ASD apps’ selection

As mentioned in Fig. 1, the second step in the SLR process includes the selection of the ASD apps to be used in our study. This step is based on a set of inclusion and exclusion criteria.

Below, we present the inclusion criteria (IC). These criteria were linked using the operator AND. This means that all the selected apps respect all the IC.

- **IC1:** Free or paid apps for iPhone or Android devices available in the Apple App store or Google Play store, respectively.
- **IC2:** Apps addressed to autistic adults (college students or workers) as primary users.
- **IC3:** Apps that are ranked 4+ stars.

Below, we present the exclusion criteria (EC). Each app that includes one of the EC will be excluded.

- **EC1:** Apps without English or French interface.
- **EC2:** Duplicate apps; available in both stores, are considered as one app.

**Figure 2:**
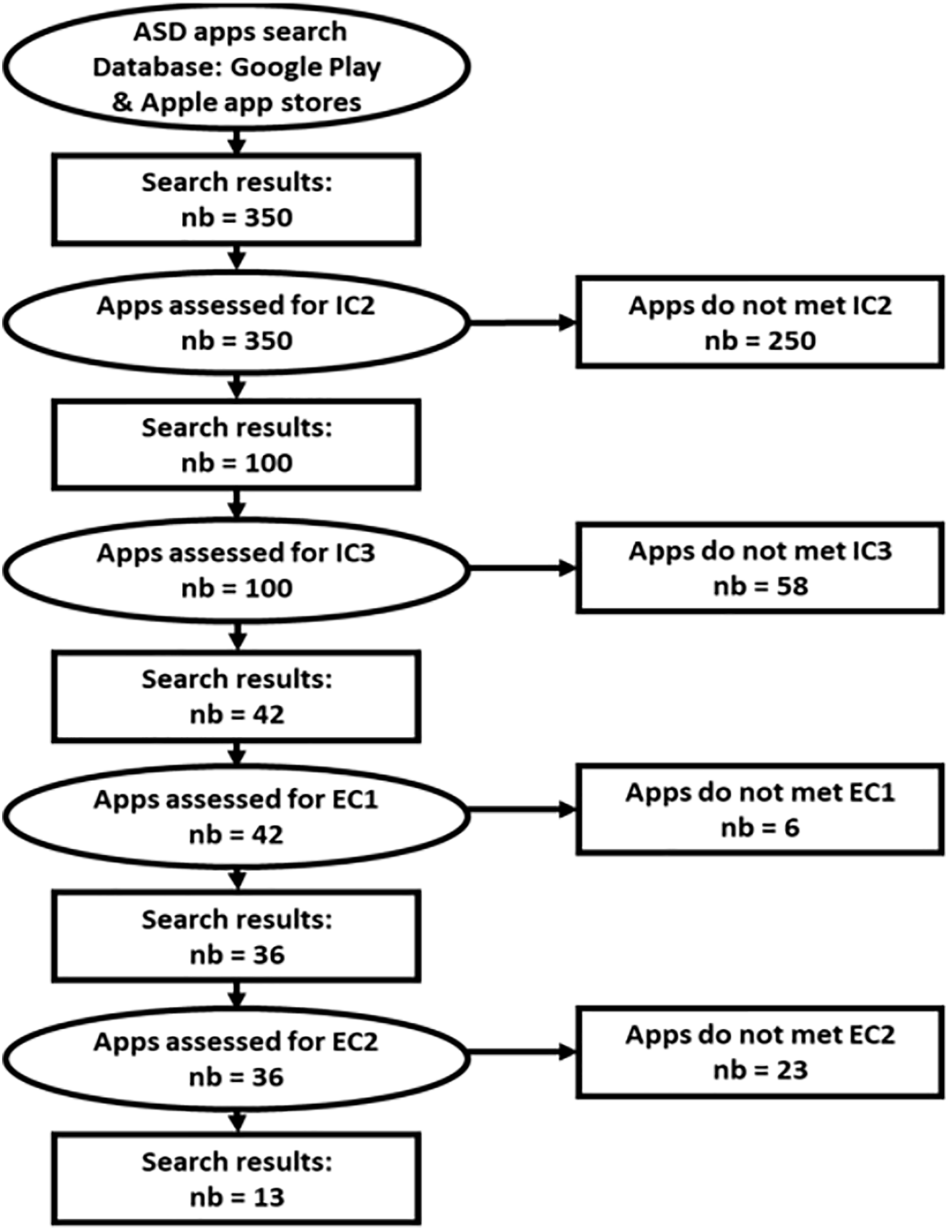
Representation of the ASD apps selection process.

Figure 2 depicts the adapted process for ASD apps selection. The total number of the selected apps for the rest of this study is 13 ASD apps. Note that the initial number of the collected apps is 350. By applying IC2, we eliminated 250 apps. Hence, we kept 100 apps after applying IC2. In fact, the majority of ASD apps available on Apple App and Google Play stores are dedicated for children for several objectives such as education (*e.g*., Rhyming Words, Autism ABC App, *etc*.), communication (*e.g*., Let’s be Social—Social Skills Development, SocialSkills for Autism Kloog2, *etc*.), *etc*. In addition, some ASD apps are used by parents of autistic children such as Autism Parenting Magazine. By applying IC3, 58 apps are eliminated. Hence, we kept 42 ASD apps. Using the exclusion criteria, we excluded six apps because they do not have English or French interfaces. After applying EC1, we kept 36 apps assessed for EC2. Using EC2, we excluded 23 duplicated apps. By applying all the inclusion and exclusion criteria, our final set of ASD apps consists of 13 apps.

In order to answer the main objective of this article, we consider only the mobile apps that are relevant to adults (college students and employees) with ASD.

## Machine learning and deep learning for user feedback analysis

In the second contribution of this article, we analyze the collected user feedback about the selected 13 ASD apps on applying both the deep learning (DL) and machine learning (ML) models to classify them into positive, negative, or neutral opinions. In this section, we detail the different steps applied for sentiment analysis of user feedback on ASD apps in order to improve the quality of life for autistic students/workers using ML and DL.

### Dataset construction and data cleaning

In order to construct our dataset, we firstly searched for the ASD apps using the keywords “autism spectrum disorder”, and “autism” on Google Play and Apple App stores. Then, to select the most relevant ASD apps, we analyze user reviews suggested on highly ranked apps for college students, and workers with autism, hence, a total of 206,885 reviews have been collected from 13 ASD apps (see Table 1).

**Table 1:** The number of user reviews collected from the 13 selected apps.

ASD apps	Rank	Number of user reviews	Number of downloads
HabitRPG	4.3	5,430	1M+
Routinely	4.8	5,430	1M+
Todoist	4.5	5,429	10M+
Work autonomy	5	5,431	
T2 mood tracker	4.7	5,430	
Miracle modus	4.4	5,430	50K+
Relax melodies	4.5	5,430	
Headspace	4.3	60,494	10M+
Honorable mention: zombies, run	4.7	393	
I can’t wake up!	4.0	22,356	5M+
Meetup.com	4.5	28,544	10M+
Mint.com	4.4	28,544	10 M+
Lyft	4.9	28,544	50M+

The data-cleaning step includes cleaning and preparing the raw data before the processing and analysis processes. The data-cleaning step include two main phases:

- Phase 1: remove the non-relevant information that has null values. Around 49.38% of the reviews were removed. Thus, the total number of reviews kept is 104,732.
- Phase 2: remove the duplicate reviews since they affect the models’ performance, around 7.33% of the reviews were removed, hence, the total number of reviews after removing duplicated ones is 97,051.

### Dataset preprocessing

The preprocessing step is a important step when using machine learning and deep learning techniques. This step encompasses the natural language processing (NLP) technique, which aims to analyze the text. In our article, this step is achieved automatically *via* the Natural Language Toolkit library (NLTK) and includes the following phases: (i) remove punctuation and special characters, (ii) remove stop words, and (iii) tokenize sentences (*i.e*., split reviews into tokens).

### Sentiment annotation/identification

Once the preprocessing step was completed, we used NLP field-based sentiment to classify the user opinion reviews into three classes: positive, neutral, and negative according to the text polarity. Sentiment analysis focuses on the polarity of a text. Based on the particular text *corpus* (wordnet), we detect the polarity and subjectivity values of the text, and based on the obtained values by applying some rules, we obtain the sentiment as positive, negative, or neutral.

Table 2 presents the total number of reviews and their classification into positive, negative, and neutral classes. As provided in this table, the total number of reviews is 97,051, where 67,278 are positive reviews, 12,460 are negative reviews, and 17,313 are neutral reviews.

**Table 2:** The user feedback (reviews) sentiment classes’ distribution.

Sentiment type	Positive	Negative	Neutral	Total
Number of reviews	67,278	12,460	17,313	97,051

### Data vectorization

In the data vectorization, we keep the unique clean tokens from the extracted reviews. Then, we applied the Term Frequency-Inverse Document Frequency (TF-IDF) vectorizer to assign a weight for each term (Ramos, 2003). This step is applied only for ML models and implemented by the sklearn library using various parameters, as illustrated in Table 3.

**Table 3:** Used parameters for term frequency-inverse document frequency.

Parameters	Values
$max\_df$	0.1
$sublinear\_tf$	True
$min\_df$	3
Norm	l2
Encoding	latin-1
$ngram\_range$	(1,3)
Lowercase	False
$use\_idf$	False

### Machine learning and deep learning models

In this section, we used six ML models such as logistic regression (LR), stochastic gradient descent (SGD), support vector machine (SVM), K-nearest neighbors (KNN), decision tree (DT), and multinomial naïve Bayes (MNB) and we investigated the use of deep learning models such as recurrent neural network (RNN), long short term memory (LSTM), gated recurrent unit (GRU). Then, we proposed to combine RNN with LSTM (RNN-LSTM) models firstly, and RNN with GRU (RNN-GRU) secondly with some improvements to classify user reviews into negative, neutral or positive opinions.

### Machine learning models

We address the classification challenge using some ML classifiers used in the literature (*e.g*., LR, SGD, SVM, KNN, DT, and MNB). These six classifiers have been chosen since they showed efficiency in recent studies for sentiment analysis (*cf.*, Oyebode, Alqahtani & Orji, 2020; Wang, Sun & Chen, 2019, *etc*.). The brief description of these chosen classifiers will be presented below.

#### Logistic regression

The logistic regression model is an essential classification technique, it is fast and simple, and it is convenient for results interpretation. This model can predict a dependent data variable by analyzing the relationship between one existing independent variable or more.

#### Stochastic gradient descent

The stochastic gradient descent model is an optimization algorithm usually used in machine learning by minimizing the cost function to find the model parameters that best fit between actual and predicted outputs. It iteratively makes small modifications to a machine learning network configuration to decrease the errors of the network. In our study, after some empirical experiments, we used the following parameters: *lossfunction* = “*modified_hube*”, penalty (regularization term) = “11”, and the number of iterations: $max\_iter = 3$.

#### Support vector machine

The support vector machine model is one of the most popular and trendy algorithms in modern machine learning proposed by Vapnik in 1995. It is a linear algorithm efficient for both regression and classification. Basically, SVM is a considered as the presentation of different classes in a hyperplane in multi-dimensional space. A space generation step is conducted iteratively by SVM when the error can be reduced.

The objective of SVM is to separate the data into several classes to find a maximum marginal hyperplane. Various kernel functions are conducted to identify new characteristic set and find the optimal output such as linear kernel function (LKF), radial basis function (RBF), and polynomial kernel function (PKF).

In this article, we used the support vector classifier (SVC) with linear LKF function, and we applied various hyper-parameters such as the penalty factor $C \in \{ 1,10,100\}$. The parameter that is the best $(C = 10)$ is chosen empirically.

#### K-nearest neighbors

K-nearest neighbors is one of the most basic yet primary classification algorithms in machine learning. It is a supervised learning technique that is applied in pattern recognition, intrusion detection, and data mining. The algorithm performs by evaluating how a data set is similar to a member of one set or the other depending on which set the data points are closest to, by searching the full data set for the number k of most comparable states, or neighbors. In our experimental, after testing several experiments for the value of k like $k = 1,3,5$, we keep k equal to 3.

#### Decision tree

The decision tree can be used for either categorical or continuous input and out-put variables. Decision trees can be generated through an algorithmic approach that can segment the dataset into different manners based on several constraints. It allows an individual or organization to weigh potential actions against one another based on their costs, probabilities, and benefits.

#### Multinomial naïve Bayes

Multinomial naïve Bayes is an approach from the naïve Bayes algorithm (which is one MNB approach). It is one of the most common algorithms, especially in NLP and text classification, it tends to do very well and show significant results. Using the Bayes theorem, the MNB classifier focuses on making probabilistic predictions of the class target given some noted features. This algorithm assumes that the attributes are independent even if they are related to each other, so it measures the conditional probability of happening for two or more events by calculating the probability of occurring for each individual event. The used parameters details for each supervised machine learning algorithm is explained in Table 4. These parameters have been chosen after several experiments.

**Table 4:** Parameters used for the used machine learning classifiers.

Classifiers	Parameters
LR	Default parameters
SGD	*lossfunction* = “*modified_hube*”, penalty (*regularizationterm*) = “*l*1”,
	Number of iterations: $max\_iter = 3$
SVM	Support vector classifier (SVC) with LKF function, $C = 10$
KNN	Euclidean distance and $k = 3$
DT	Default parameters
MNB	Default ( $alpha = 1.0$)

### Deep learning models

Using deep learning in the classification of user reviews into negative, neutral or positive opinions has become a very active research area in recent years. In this section, we investigate the use of deep learning models such as recurrent neural network (RNN), gated recurrent unit (GRU), long short-term memory (LSTM), and we suggested to combine RNN and LSTM (RNN-LSTM) models firstly and RNN with GRU (RNN-GRU) secondly with some amelioration.

In the subsections below, we will briefly describe each deep learning model and the applied modifications.

#### Recurrent neural network

**Figure 3:**
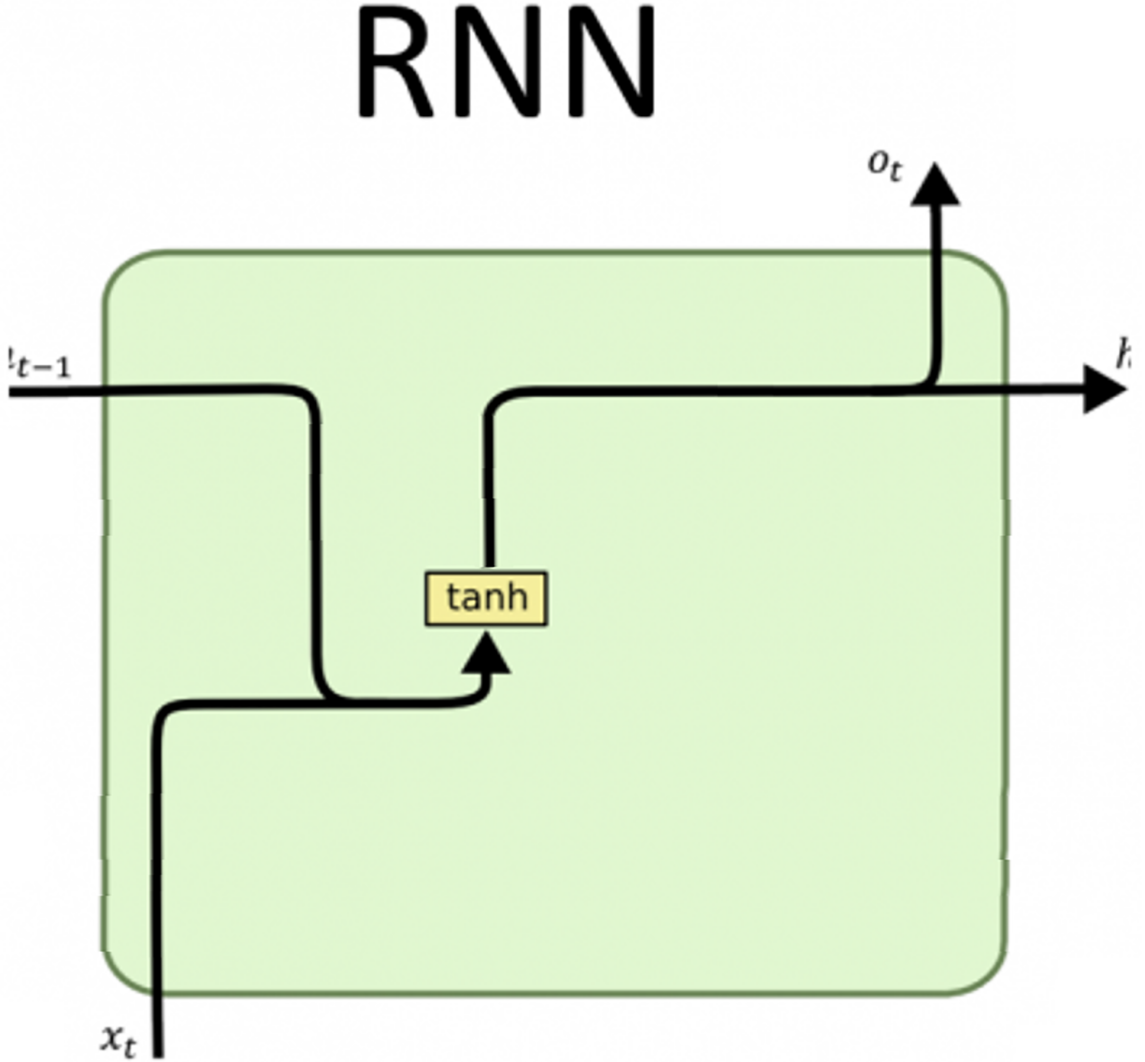
Simple RNN architecture.

Simple RNN is a strong deep neural network. It has a memory with a restricted capacity, unlike traditional feed-forward neural networks. The current input at a given time step depends on the prior inputs as well (Wazery, Mohammed & Houssein, 2018). Every simple RNN cell takes the current input ${x_t}$ and the past hidden state, which consists of information from the past ${h_{t - 1}}$, joins them and applies an activation function to it to get a value ${h_t}$, which is passed to the next simple RNN cell and used to predict the output ${O_t}$ (see Fig. 3).

#### Long short-term memory

**Figure 4:**
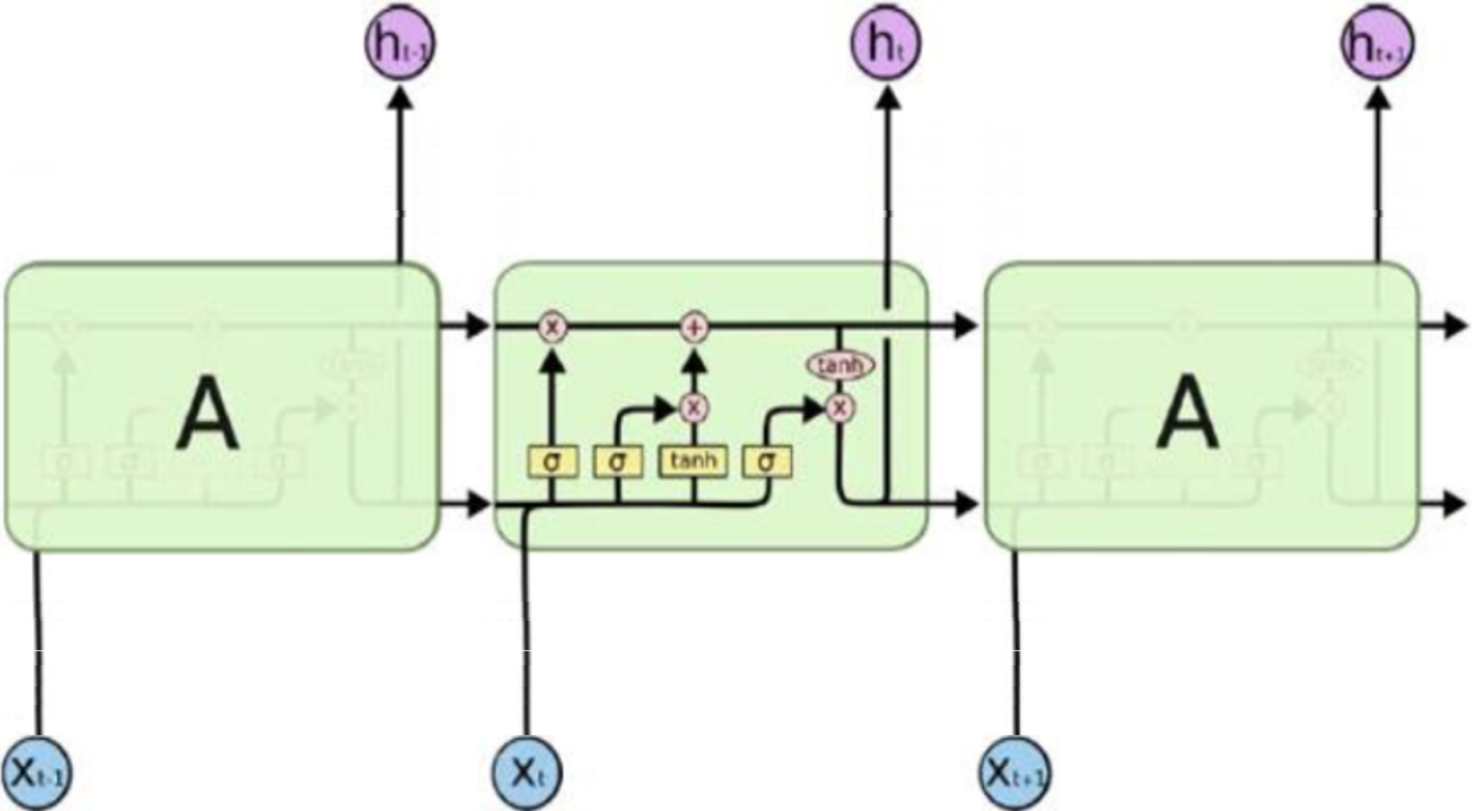
LSTM architecture.

Long short-term memory, is an RNN with an additional internal memory cell. LSTM has three gates, namely Input, Output, and Forget gate, each gate indicates the controller of an information flow. The workflow of LSTM is that, as new sequential data is fed, the memory cell is updated by adding new data and partially forgetting existing data. For each timestep $t$, all three gates are presented with the input ${x_t}$ (one element of the input sequence) along with the output h $_{t - 1}$ of the memory cells at the previous timestep $t - 1$ (see Fig. 4).

#### Gated recurrent unit

The gated recurrent unit was proposed as a simpler structure alternative to the LSTM. It is a type of gated RNN which is used to solve the common issues of vanishing and exploding gradients in classical RNNs when learning long-term dependencies (Shen et al., 2018). Compared to LSTM, GRU has only two gates: the reset gate and update gate and does not have a memory cell (Mukherjee et al., 2019).

**Figure 5:**
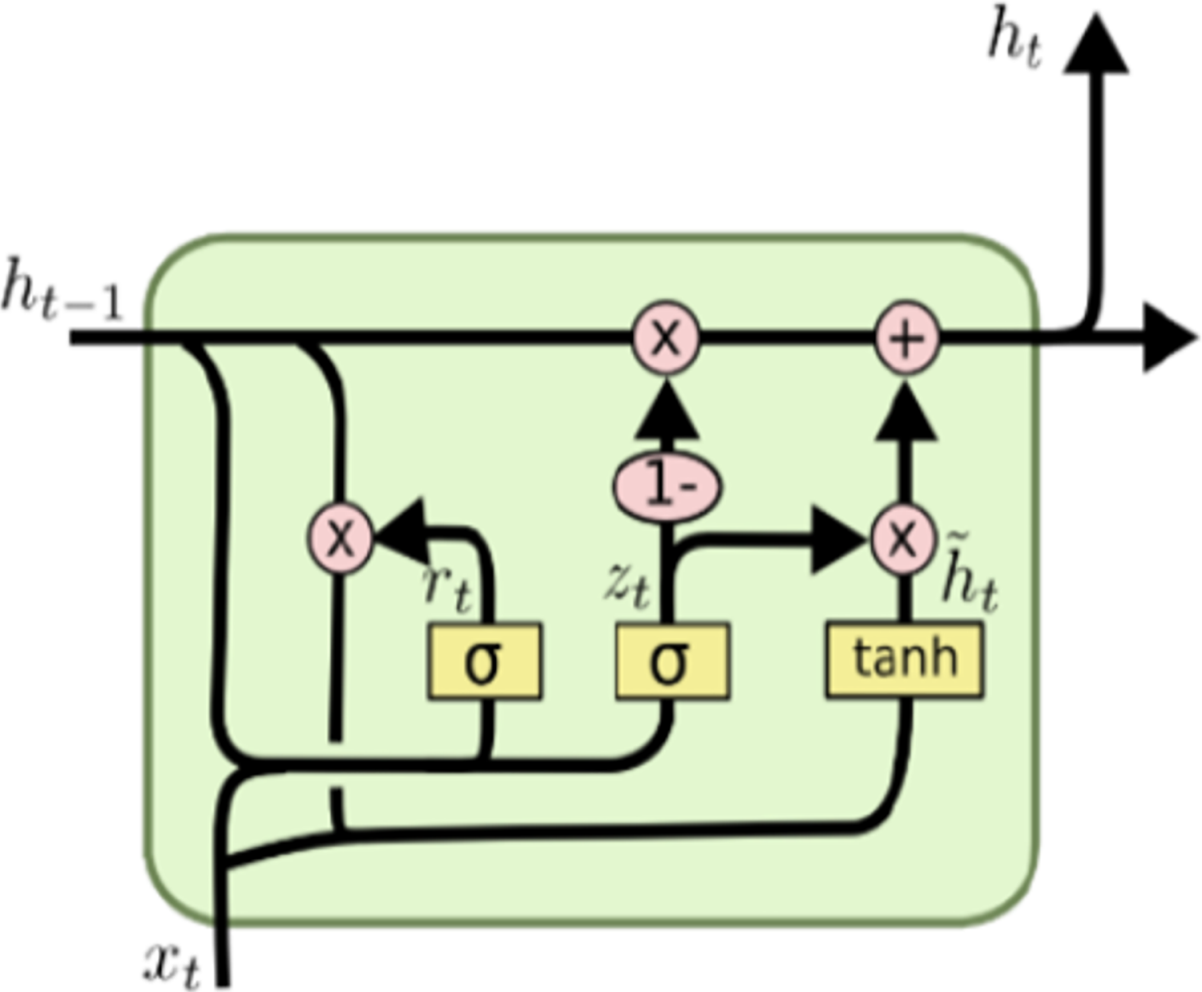
GRU architecture.

Both gates are related to two components: ${x_t}$ and ${h_{t - 1}}$. The previous comes from the input sequence, the final is the output value of memory cells from the previous time point (see Fig. 5). Therefore, each gate achieves different tasks to fulfill the purpose of filtering.

#### Proposed deep learning models

As mentioned earlier, to establish the best performing model for user feedback analysis, we investigate the use of DL models such as RNN, LSTM, and GRU, and we propose to combine RNN with LSTM firstly, and RNN with GRU secondly with some improvements. For the deep learning architecture using RNN, LSTM, or GRU model, we firstly keep an embedding layer with an input dimension of the size of the data vocabularies, and an output dimension of 1,000 words, secondly, we used one recurrent layer (RNN, LSTM, or GRU) with 128 units, followed by three dropout layers of 0.9, 0.09 and 0.05, respectively. This dropout layers are performed to ameliorate this architecture by decreasing the over-fitting problem. Finally, one dense output layer with the softmax activation function is applied for the classification problem. Then, to increase the learning rate, we adapted the root mean square prop (RMSprop) optimizer.

**Figure 6:**
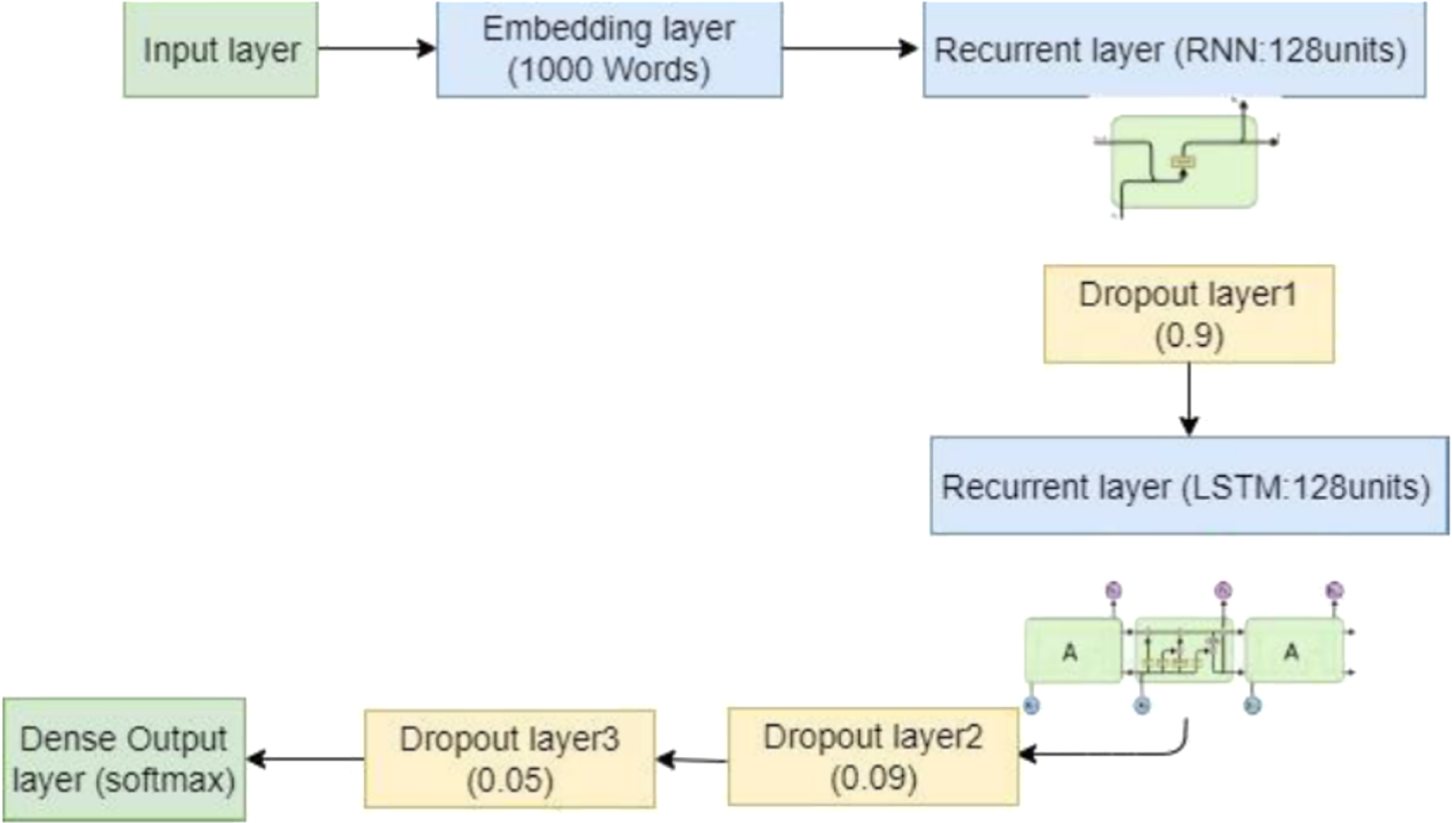
The architecture of the proposed deep model RNN-LSTM.

In addition, for the combination of DL architectures (RNN-LSTM, and RNN-GRU), we started with an embedding layer (When the input dimension of the size of the data vocabularies, and the output dimension of 1,000 words). In RNN-LSTM, we implemented two recurrent layers; the first layer is the RNN layer with 128 units is conducted followed by one dropout layer of 0.9, which is conducted to ameliorate this architecture by decreasing the over-fitting issue. The second layer is the LSTM layer with 128 units, followed by two dropout layers of 0.09 and 0.05. For RNN-GRU, we also implemented two recurrent layers, the first recurrent layer is the RNN layer and the second is the GRU layer with the same unit number and dropout layer values compared to the RNN-LSTM model. Finally, one dense output layer in which data are classified into three categories positive, negative, and neutral using the softmax function as an activation function. Then, we adapted the RMSprop optimizer to rise the learning rate. Figure 6 presents our proposed DL architecture (RNN-LSTM) with an embedding layer, one recurrent layer (RNN); one dropout layer; one recurrent layer (LSTM), two dropout layers, and one dense output layer.

In the training part, we take the RMSprop optimization to increase the learning rate, 20 epochs, and batch size equal to 90 with the loss function “categorical cross-entropy” as training parameters. The details of all used training parameters in the RNN-LSTM model are shown in Table 5.

**Table 5:** Parameters used for deep learning models.

Parameters	Values
Embedding ( $input\_dim$)	Total number of words
Embedding ( $output\_dim$)	1,000
Unit	128
$Dropout\_1$	0.9
$Dropout\_2$	0.09
$Dropout\_3$	0.05
Batch size	90
Epochs	20
Activation function for output layer	Softmax
Optimizer	RMSprop
Lowercase	False
Loss function	Categorical cross-entropy

## Experimental results

In this section, we discuss several experimental results. These experiments were conducted with Python language and on an AMD Graphic card and Intel core i5 CPU processor with 8 GB memory. We indiscriminately divided the database into 75:25 to build respectively the training and the testing sets. To assess the performance of our models, we utilized the following metrics: accuracy, precision, recall, and F1-score.

In Table 6, we offered the performance evaluations for the sentiment classification of user reviews on ASD apps using some machine learning classifiers. As it is shown in this table, LR reached the best overall accuracy of about 89.80%, followed by SVM, DT, SGD, MNB, and KNN with 89.13%, 87.45%, 85.04%, 78.56%, and 33.17% respectively.

**Table 6:** Evaluation of sentiment analysis of ASD mobile apps reviews using machine learning classifiers.

Classifiers	Accuracy	Precision	Recall	F1-score
MNB	78.56	78.24	78.56	75.83
SGD	85.04	85.18	85.04	84.05
KNN	33.17	76.53	33.17	32.85
DT	87.45	87.46	87.45	87.43
SVM	89.13	89.23	89.13	88.74
LR	89.80	89.85	89.80	89.57

**Figure 7:**
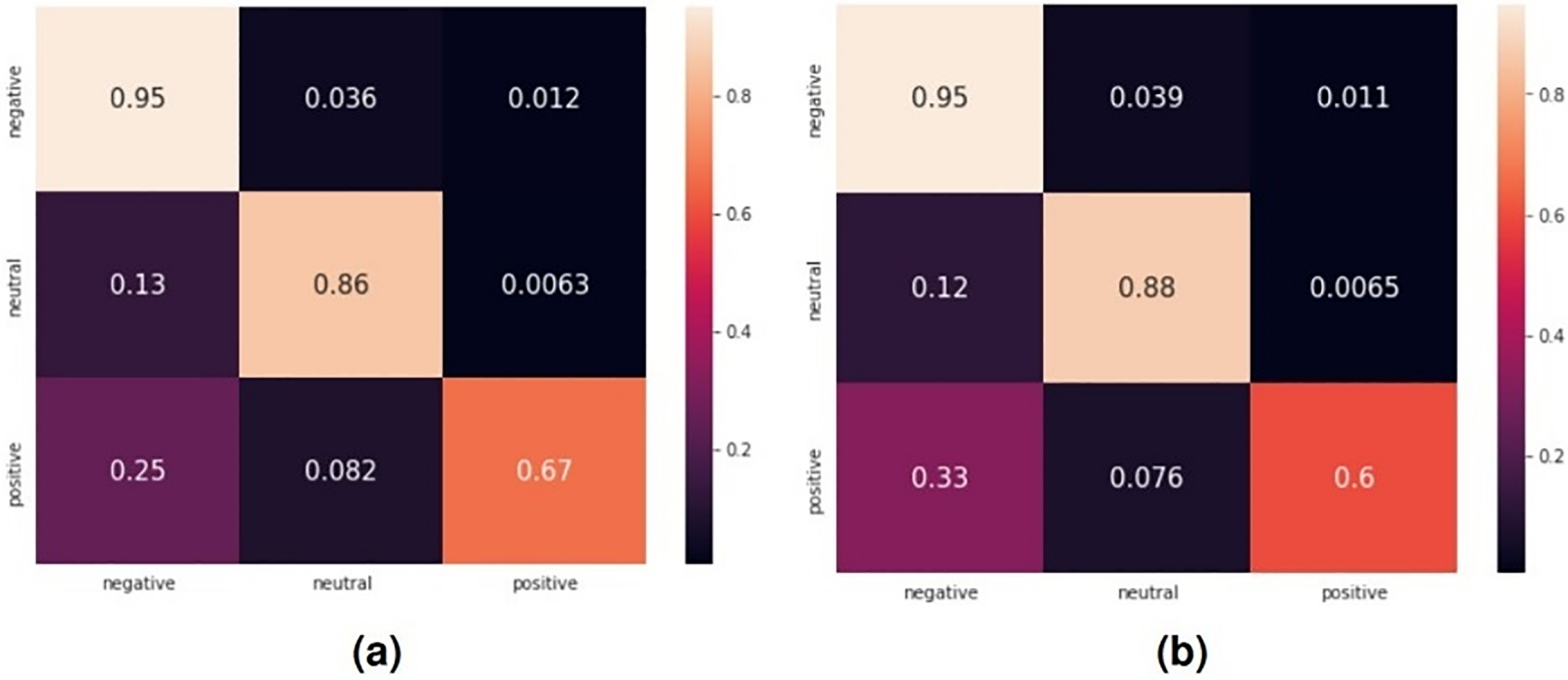
Confusion matrix for the sentiment classification of user reviews on ASD apps. (A) Confusion matrix for LR classifier, (B) confusion matrix for SVM classifier.

Moreover, as displayed in this table, we also evaluated the performance of the selected classifiers using other metrics such as precision, recall, and F1-score. For all these metrics, the LR classifier outperforms the other classifiers. Figure 7 displays the confusion matrices for the sentiment classification of user reviews on ASD apps using the LR and SVM classifiers since they achieved better results compared to other classifiers.

As it is revealed in this figure, for the confusion matrix obtained by the LR classifier, 95% of the negative reviews have been correctly classified as negative, whereas 86% of the neutral reviews have been correctly classified as neutral and 67% of the positive reviews have been correctly classified as positive. Our system often makes mistakes mostly in classifying positive reviews, where 25% are classified as negative and 8.2% are classified as neutral.

For the sentiment classification of user reviews on ASD apps using deep learning models, we conducted a set of experiments, which have been performed over three famous deep learning models such as RNN, LSTM, GRU, and the two proposed models RNN-LSTM and RNN-GRU. These results are obtained by applying 20 Epochs, and batch size equal to 90. Table 7 reveals the obtained performances from the three deep learning models (RNN, LSTM, and GRU), and two proposed models RNN-LSTM and RNN-GRU.

**Table 7:** Evaluation of sentiment analysis of user reviews on ASD apps using deep learning models.

Model	Accuracy (%)	AUC (%)
RNN	95.19	98.82
LSTM	95.88	99.19
GRU	95.55	99.10
RNN-LSTM	96.58	99.41
RNN-GRU	95.92	99.21

As illustrated in Table 7, the best results have been provided by the RNN-LSTM model as two recurrent layers with an accuracy of 96.58% and an AUC of 99.41%. Followed by the RNN-GRU (Accuracy = 95.92%; AUC = 99.21%), LSTM (Accuracy = 95.88%; AUC = 99.19%), GRU (Accuracy = 95.55%; AUC = 99.10%), and RNN (Accuracy = 95.19%; AUC = 98.82%).

**Figure 8:**
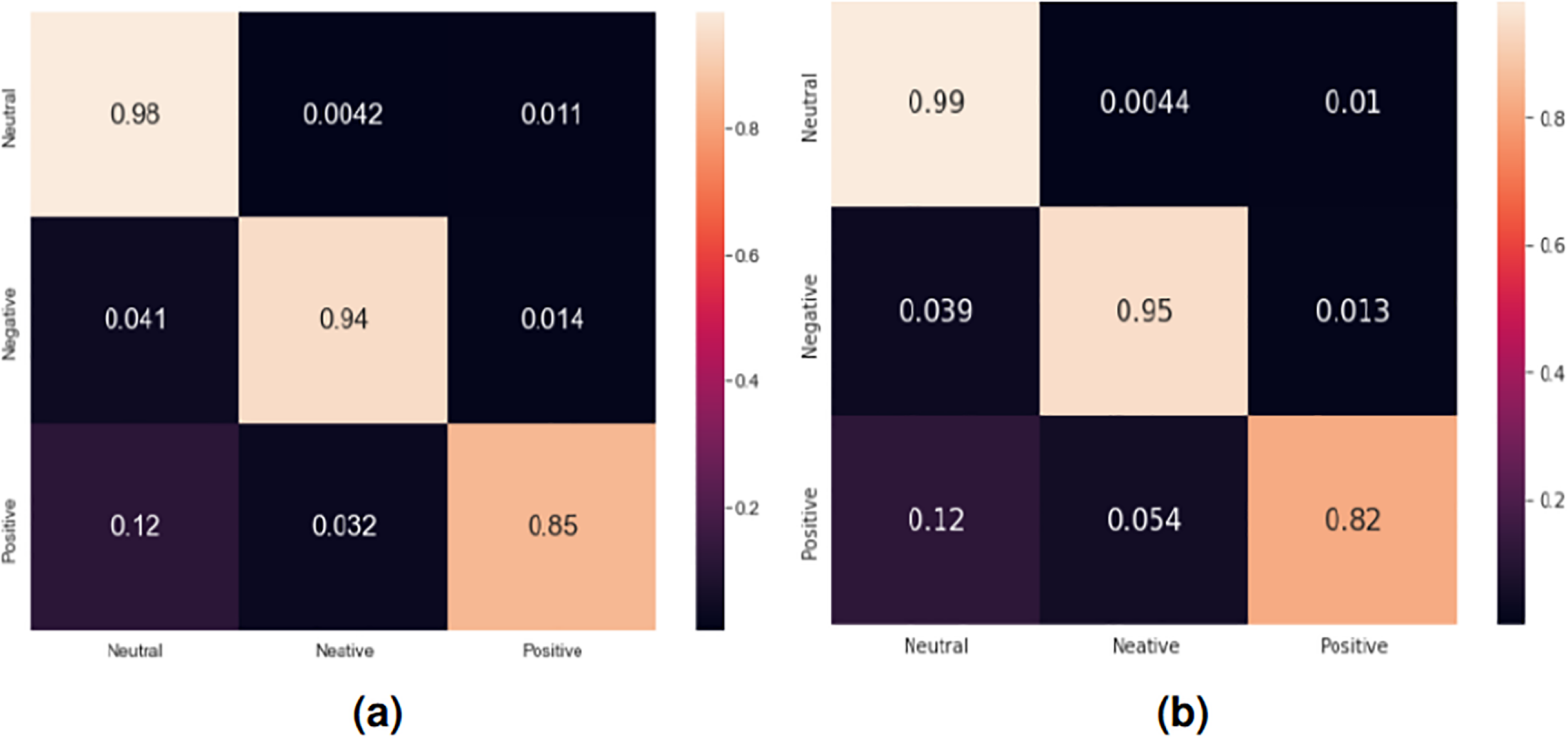
Confusion matrix for the sentiment classification of user reviews on ASD apps. (A) Confusion matrix for RNN-LSTM model, (B) confusion matrix for RNN-GRU model.

Figure 8 presents the confusion matrices for the sentiment classification of user reviews on ASD apps using the RNN-LSTM and RNN-GRU models since they achieved better results compared to other deep learning models.

As revealed in this figure, regarding the confusion matrix obtained for the RNN-LSTM model, 98% of the neutral reviews have been correctly classified as neutral, whereas, 94% of the negative reviews have been correctly classified as negative and 85% of the positive reviews have been correctly classified as positive. Our system often makes mistakes mostly in classifying positive reviews, where 12% are classified as neutral and 3.2% are classified as negative.

In addition, we applied several experiments in our training model with different Epoch numbers such as 10, 20, 30, and 40. In order to validate the choice of epoch’s number. Table 8 addresses the effects of varying the number of epochs on the RNN-LSTM performances. These experiments showed that the best results are provided with 20 epochs.

**Table 8:** Results with Epoch number variation using deep learning model (RNN-LSTM).

Epoch number	10	20	30	40
RNN-LSTM	99.20	99.41	99.35	99.35

## Discussion

### Challenges and recommendations for ASD apps

The features provided by ASD apps for college students and workers are identified in this study. In fact, previous research studies (*cf.*, Rehman et al., 2021; Gallardo-Montes, Caurcel Cara & Rodríguez Fuentes, 2022, *etc*.) focused on ASD apps for children, however, college students and workers with ASD might experience several communication issues, which make their daily activities challenging. ASD apps provide a great opportunity for them in improving their skills. Moreover, the environment influences people with ASD behavior such as loud noises, strong smells, *etc*. The challenges mentioned in the previous research studies (*cf.*, Sulaimani & Gut, 2019; Bury et al., 2021, *etc*.) address mainly the difficulty to access the several provided services, the number of individuals for each service, limited knowledge about autism, *etc*. In addition, recent statistics proved that around 85% of the United States college graduates with autism are unemployed (Catalano.N, 2020). Autistic workers confront several challenges in their workplace such as the lack of accessibility to appropriate employment resources, difficulties in adjusting to the new environment, *etc*. In order to determine whether the ASD apps available on Google Play and Apple App stores satisfy the needs of autistic adults, we investigated the user feedback on ASD apps. This analysis will allow us to propose some lines for apps’ improvements in the near future to improve the negative features in order to better satisfy the autistic needs. Several ASD apps used AI-based techniques for speech assistant (*e.g*., speech translation, speech to text, *etc*.), the use of ASD apps could improve autistic’s life especially those with communication difficulties.

As we mentioned previously, user reviews have been classified into positive, negative or neutral. All these categories provide user opinions towards the ASD apps or towards a specific feature offered by them. By analyzing positive reviews, we noticed that ASD apps help users successfully manage their daily activities, track their tasks, and ensure appropriate communication with others. For instance, the users of the HabitRPG app considered that *“This app has definitely helped me get my life back on track”*. ASD apps are recommended for people with autism to develop their social interactions. In fact, autistic in general have deep difficulties to communicate with their colleagues in universities, work space, *etc*. Providing several ways for communication is very helpful in this situation.

By analyzing negative reviews, we noticed that usually users complained about the quality of ASD apps (*e.g*., availability, usability, *etc*.). For instance, users requested that the app should work offline *“Can you make this Offline”*, and *“I just want to re-quest an update that would keep me tracking my habits and dailies even when I am offline”*. While others criticized the complexity of some interfaces *“It’s a nice concept, but there’s too much information given at once for setting it all up”*, *“Good app but it is confusing”*, and *“It looks super good but I can’t figure it out how to use it”*. Mobile apps for ASD people should not be complex. In fact, sometimes, the quality factor of ASD apps is classified as sophisticated and advanced functionality. It is not critical or compulsory for a patient’s health but it facilitates and attracts users. Users also revealed other quality issues such as portability issues such as *“I can’t login to android version or register on it”*, and *“It’s a very good app if you want build need habits”*, reliability issues *“It crashes”*, and maintainability issues *“As soon as I did the update my acct. was gone. What happened?”* In addition, other users complained about the sounds used in some ASD apps such as *“I only tried it for about 10 minutes but I hated the sounds. That’s the main thing I wanted”*. In fact, noise may affect autistic and increase their anxiety. For these reasons, developers should work on improving the quality of ASD apps.

Besides the quality improvement, some users suggested to update existing functionality. For instance, for the “Todoist” app, users proposed to update the set up a task feature by allowing the user to add a task by indicating only the week and not the day *“There’s no good way to set up a task that you want to do once a week but on any day”*, and *“Good for people who want to develop daily habits. Not so good for weekly habits”*. In fact, the calendar feature is very important for people with ASD to help them managing their daily tasks as well as their habits. This feature has been mentioned by many users of the selected apps such as *“There is no sort of calendar overview”*. In addition, daily tasks must be related to weekly habits. Users recommended to add connect the daily tasks with the habits in order to check the user performance history, *“Allows me to track dailies, but doesn’t connect them to habits”*. Actually, people with autism have a resistance to change. Hence, having and maintaining a routine is very important for autistic. Other users revealed an issue about the notifications feature in some apps such as “Todoist” and “HabitRPG” for instance *“Notifications don’t work”*, *“I need the notifications and they have NEVER worked for me*, *etc*.”, *“I’d rate 5 stars if the app didn’t send reminders for stuff I’ve done already”*, and *“Sure it gamifies your tasks, but it doesn’t remind you of anything”*. In fact, notifications is an important feature for tasks management, it is very important to reminder the user about his activity. In addition, users suggested to add sub-task list in the “Todoist” app *“Wish there is a way to add sub-lists under one task”*. On the other hand, users complained about some features that may not work correctly in their country such as registration payment for instance, *“Kindly create an MPESA payment method?”*. Moreover, users have reported some bugs such as The habit counter is always resetting itself. Which means the feature exists but it includes some mistakes that must be corrected. A habit counter should not be reset unless the user asked for it. Finally, the users were not satisfied by the registration feature in some apps for example *“I can’t register”*, and *“Please help me Wanted to delete my account. There is no option to do so”*.

In summary, to answer the first research question, we conclude that technologies such as smartphones and ASD apps can be beneficial in helping adult autistic manage their daily activities and improve their communication skills with their colleagues, supervisors, *etc*. In addition, ASD apps are used by ASD people to better organize their tasks. Regarding the second research question, we conclude that despite their satisfaction with ASD apps, users proposed adding some features such as statistics about daily activities and the to-do list. These features have been highly requested by ASD apps users. In addition, the use of notification is the most requested feature by users, while others are complaining about the reminder sound. Based on the negative reviews, mobile apps developers should improve the quality of ASD apps and add the requested features (*e.g*., notification, daily activities, *etc*.). In addition to the quality characteristics, some functionality in the selected apps should be improved as well. In fact, although several apps provide the management tasks feature, users are not really satisfied with it. In addition, the notifications feature is missing in some apps or does not work correctly. These two features are very important for people with ASD since they help them managing their lifestyle and keeping their routine.

### Limitations

In this sub-section, we discuss the limitations of this study. In fact, the analysis of the main features provided in this study is based on the feedback of users. Although, the selected ASD apps are developed mainly for people with ASD (*e.g*., Work Autonomy, T2 Mood Tracker, *etc*.), there is no guarantee that all the collected reviews are written by autistic people. For this reason, it is recommended for future work to test the selected ASD apps by people with autism and get their feedback instantly. blackMoreover, some reviews such as “great”, “useful”, and “Amazing”, could be considered as fake feedback. In our preprocessing tasks we did not remove them, however, these reviews do not affect the results obtained in this article. In fact, in our analysis we focus on the features provided by the ASD apps and how well the user is satisfied with it. And since these reviews do not talk about any feature, they will not be analyzed. In addition, the features provided by ASD apps should be also identified by installing and testing them.

On the other hand, each user review has been labeled with one type (positive, negative, or neutral). But in reality, one review may include more than one type. In fact, over the process of labeling reviews, we noticed that the proportion of the reviews with multiple types is low. In addition, we used a tokenization with our intervention to separate these reviews into a fine-grain sentences, which can be labeled with one type before the classification experiments are conducted. Hence, we believe that this issue did not affect our experimental results. Moreover, the dataset used in this article includes a number of positive reviews more important than the negative reviews. In order to limit the problems due to the imbalanced data, as recommended by Ito et al. (2021), we used the right evaluation metrics to evaluate the machine learning models. Hence, we were not restricted in our evaluation on the accuracy metric but also we used precision, recall, F1 score, and AUC. In fact, accuracy is not the best metric to be used in evaluating machine learning models with imbalanced datasets (Ito et al., 2021). Other metrics could be better such as: the confusion matrix, which show the correct predictions and incorrect predictions; precision, which measure the classifier’s exactness; recall, which measure the classifier’s completeness, *etc*. For this reason, we can conclude that the used machine learning models have been appropriately evaluated even with an imbalanced dataset.

## Conclusions

The main issues of ASD are (1) the inability and the deviance in social communication and interaction among the world, and (2) the limit and repetitive movements, the restriction of the patterns of behavior, interests, and the presence of stereotyped activities. The existence of these symptoms leads adults (students or workers) with ASD to have several challenges when forming new meaningful relationships. An individual with Autism has a manner to communicate either verbally, non-verbally, or both. Understanding the awareness of ASD in our community helps in improved sup-port for adults with ASD in their universities and companies.

In this article, we investigated the opinions of ASD app users to understand their perspectives on an autism-friendly environment in order to suggest possible key in-sights for the improvement of these apps. For this purpose, we applied the SLR process to select the 13 most relevant ASD apps. Then, we used ML and DL models for user feedback analysis on ASD apps, hence, the collected user reviews from the selected 13 ASD apps are classified into positive, negative, or neutral opinions. We collected 97,051 reviews including positive, negative and neutral opinions from the Google Play and Apple App stores. After that, in the process of user reviews analysis, we applied NLP techniques, ML, and DL models and we proposed to combine RNN with LSTM models firstly, and RNN with GRU models secondly, with some amelioration, in order to classify user reviews into negative, neutral, or positive opinions. The evaluation results proved that the RNN-LSTM model provided the best accuracy of 96.58% and best AUC of 99.41% in classifying user reviews according to sentiment polarity. In addition, by analyzing the collected reviews, we concluded that despite the satisfaction of ASD app users towards the main features provided by these apps, users still complain especially about their quality issues especially usability, reliability, *etc*. Developers should focus more on improving the quality of their ASD apps and adding some features such as notification and to-do-list.

For future work, we propose to classify the collected user reviews according to the quality characteristics of the ISO 25000 quality model (*e.g*., usability, reliability, portability, *etc*.). Moreover, we will work on prioritizing relevant user reviews, which could be helpful in identifying the quality characteristics that should be improved first.

## Supplemental Information

10.7717/peerj-cs.1442/supp-1 Supplemental Information 1 Clean Data.

10.7717/peerj-cs.1442/supp-2 Supplemental Information 2 Raw Data.

10.7717/peerj-cs.1442/supp-3 Supplemental Information 3 Source Code.

## Additional Information and Declarations

### Data Availability

The following information was supplied regarding data availability: The raw data and code are available in the Supplemental Files.
